# Poverty and fever vulnerability in Nigeria: a multilevel analysis

**DOI:** 10.1186/1475-2875-9-235

**Published:** 2010-08-19

**Authors:** Oyindamola B Yusuf, Babatunde W Adeoye, Oladimeji O Oladepo, David H Peters, David Bishai

**Affiliations:** 1 Department of Epidemiology and Medical Statistics, College of Medicine, University of Ibadan, Ibadan, Nigeria; 2 Department of Economics, University of Lagos, Lagos, Nigeria; 3 Future Health Systems Research Programme Consortium, Department of Health Promotion & Education, College of Medicine, University of Ibadan, Ibadan, Nigeria; 4 International Health, Health Systems programs. John Hopkins School of Public Health, Baltimore, USA; 5 Dept of Population, Family and Reproductive Health, John Hopkins School of Public Health, Baltimore, USA

## Abstract

**Background:**

Malaria remains a major public health problem in Sub Saharan Africa, where widespread poverty also contribute to the burden of the disease. This study was designed to investigate the relationship between the prevalence of childhood fever and socioeconomic factors including poverty in Nigeria, and to examine these effects at the regional levels.

**Methods:**

Determinants of fever in the last two weeks among children under five years were examined from the 25004 children records extracted from the Nigeria Demographic and Health Survey 2008 data set. A two-level random effects logistic model was fitted.

**Results:**

About 16% of children reported having fever in the two weeks preceding the survey. The prevalence of fever was highest among children from the poorest households (17%), compared to 15.8% among the middle households and lowest among the wealthiest (13%) (p<0.0001). Of the 3,110 respondents who had bed nets in their households, 506(16.3%) children had fever, while 2,604(83.7%) did not. (p=0.082). In a multilevel model adjusting for demographic variables, fever was associated with rural place of residence (OR=1.27, p<0.0001, 95% CI: 1.16, 1.41), sex of child: female (OR=0.92, p=0.022, 95% CI: 0.859, 0.988) and all age categories (>6months), whereas the effect of wealth no longer reached statistical significance.

**Conclusion:**

While, overall bednet possession was low, less fever was reported in households that possessed bednets. Malaria control strategies and interventions should be designed that will target the poor and make an impact on poverty. The mechanism through which wealth may affect malaria occurrence needs further investigation.

## Background

Malaria is one of the most important challenges to public health with about 300 to 500 million cases reported annually. More than 1 million people die from the disease, most of them children under age 5 years. Over 90.0% of the cases and 75% of the deaths occur in sub-Saharan Africa (SSA). These childhood deaths, resulting mainly from cerebral malaria and anaemia, constitute somewhere between 20% and 25% of child mortality in Africa [1,2].

African countries south of the Sahara bear the heaviest burden of malaria. These countries are among the poorest in the world and widespread poverty on the continent continues to play a role in the burden of the disease. Malaria cases and deaths have risen steadily in sub-Saharan Africa since the late 1970s, especially in Nigeria. The emergence of resistance to insecticides and chloroquine, the cheap anti-malarial treatment widely used for clinical management of uncomplicated malaria, has been held as a major factor in this trend, aided by a general weakening of health systems. This effect was exacerbated by economic stagnation and decline, which has implications for growth and welfare. For instance, malaria is responsible for about a 1.3 per cent reduction in the average annual rate of economic growth for those countries with the highest burden. In Nigeria, malaria is the major cause of morbidity and mortality, especially among children below age five [3]. Malaria is a social and economic problem, which consumes about US$3.5 million in government funding and US$2.3 million from other stakeholders in the form of various control attempts in 2003[4].

Given the above scenarios, the drive of the government to achieve the targets of the National Economic Empowerment and Development Strategy (NEEDS), Seven-Point Agenda and the MDGs is currently threatened by the seemingly intractable trend of the ancient scourge which has compounded national and household poverty through intensive loss of productive time to attack and death in extreme cases. This is compounded by the development of resistance to the traditional first-line drug (chloroquine), while the newly introduced therapy (ACT) would be largely beyond the reach of many due to poverty, if it was not heavily sponsored by international organizations. Given that malaria is endemic throughout Nigeria, and that over 50.0% of the country's population are living below poverty line, malaria incidence may increase significantly in Nigeria because many may not be able to afford the newly introduced drugs due to poverty.

There are quite a good number of attempts to analyse the economic effects of malaria in the literature [2,3,5-7]. Most of the studies are limited to determining the mathematical significance of malaria. However, a comprehensive analysis of malaria and poverty remains scarce. The links between malaria and poverty are multiple and complex. Therefore a better understanding of the direction and magnitude of the causal relationship is needed, along with better understanding of the nature of poverty that is related to malaria. For example, understanding whether the relationship between malaria and poverty is related to household factors, community, or larger regional factors would help to identify whether further investigation and action is needed at one or more of these levels. Poverty sustains the conditions where malaria thrives, and malaria impedes economic growth and keeps communities in poverty. With a potential dual relationship between poverty and malaria, where poor households experience high malaria prevalence that in turn maintains them in poverty, these households are trapped in reinforcing cycles [8,2].

There are many pathways through which the relationship between malaria and poverty operates. At the household level, poor housing can expose people to contact with infective mosquitoes. Simple preventive measures such as insecticide-treated bed nets are unaffordable to the poorest if they must pay for them. Lack of resources prevents people from seeking timely health care. Often, peak malaria transmission coincides with harvesting time, which is a factor that affects risks at the village level. A few days of work lost to illness can mean food insecurity for the entire family.

A meaningful relationship between malaria and poverty should consider the effect of both individual and cluster where individual belongs. This approach requires a multilevel analysis incorporating variables at different levels of aggregation. In this report, the relationship between poverty and malaria in Nigeria was analyzed using a multi-level logistic model. The independent variables considered were bed net ownership, type of place of residence, sex and age of child, and literacy level of mother.

## Methods

This report utilizes secondary data from the Nigerian Demographic and Health Survey [9]. Demographic and health surveys are an international series of nationally representative surveys conducted in middle and lower income countries [10].

The study population in this analysis was the 25,004 under five children extracted from the records of 104,808 respondents who responded to the household questionnaire. The outcome measure in this analysis was occurrence of fever in children less than five years of age in the last two weeks preceding the survey. The explanatory variables were sex of child, age of child, whether or not household possess a bed net, whether child slept under bed net night before survey, type of bed net (treated or untreated), type of place of residence (rural/urban) and wealth index; a measure of poverty. This index serves as a proxy for measuring the long-term standard of living. It was based on data from the household's ownership of consumer goods; dwelling characteristics; type of drinking water source; toilet facilities and other characteristics that are related to a household socio-economic status such as car, radio, television, gas cooker, iron, boat/ship, telephone, source of water, electricity supplies, and type of wall, floor and roof, etc. To construct the index, each of these assets was assigned a weight (factor score) generated through principal component analysis, and the resulting asset scores were standardized in relation to a standard normal distribution with a mean of zero and standard deviation of one [11]. Each household was then assigned a score for each asset, and the scores were summed for each household. Individuals were ranked according to the total score of the household in which they resided. The sample was then divided into quintiles from one (lowest) to five (highest). A single asset index was developed on the basis of data from the entire country sample and this index was used in the analysis. The data were analyzed using Stata 10.0 [12].

Frequency tables were generated for relevant variables. Descriptive statistics such as means, medians and standard deviations were used to summarize continuous variables. Relationships between the outcome measure (occurrence of fever) and each explanatory variable was first explored using the chi squared test in bivariable analysis before a multilevel logistic regression was carried out. Explanatory variables found to be associated with the outcome were included in the logistic model based on the magnitude of the chi squared values. Odd ratios and 95% CIs were computed. A random effect logistic model was fitted that included fixed effects and group-level intercepts as random effects [13]. This allowed for a two-level, inherently nested nature of the data: individuals (children) nested within clusters/regions. Nigeria has six geopolitical zones and there is variation in all characteristics including fever patterns in all the zones.

Multi-level models allow the additional information provided by knowing which cluster a child comes from to be taken into account in modeling the relationship between independent and dependent variables. This model was run using the GLLAMM routine in Stata. In using this approach, the correlation between children from the same cluster or region arises from their sharing specific but unobserved properties of the respective regions. A random effect logistic regression model for the data is given by

Logit (Pr(Yij=1|Xij,Ui)=b0+b1child'sage+b2type of place of residence+b3sex+b4wealth index+UiUi~N (0,τ2).

Where b_1, _b_2, _b_3 and _b_4 _represents fixed effects and U_i _represents a random effect. U_i _is a random variable with a mean of zero and constant variance. U_i, _is the estimation of the variance across all of the regions involved in the study. If the variance is large then the outcome of interest is dependent on the region, if the variance is small then the variations in outcome of interest may be explained by the measured characteristics alone.

Given U_i, _the responses from the same regions are mutually independent that is the correlation between children from the same regions is completely explained by them having been observed in the same regions. The variance τ^2 ^measures the degree of heterogeneity in the probability of experiencing fever that cannot be explained by the classification into the 2 categories (fever: yes Vs no)

An important measure that describes these dependences in the data is called the intra-class correlation coefficient (ICC); this statistic measures the extent to which individuals within the same group are more similar to each other than they are to individuals in different groups. The intraclass correlation (ICC), ρ was calculated using:

$$\rho =\frac{\tau }{\tau +\pi^{2}/3}$$

Where **τ = **estimated variance and π = 3.142

### The Gllamm approach

This is a stata procedure for fitting generalized linear models, a class of regression models for univariate responses with density from an exponential family [14]. The logistic regression model was specified from the family of generalized linear model with the logit link and binomial distribution using the link ( ) and family ( ) options. The option i(region) specifies the desired within region correlation matrix. The eform produces the estimated odds ratios. The full syntax is given below:

xi: gllamm fever i.place of residence i.sex of child i.childsage i.wealthnw, i(region ) family(binomial) link(logit) adapt nip(5), eform

## Results

### Characteristics of the sample

The study sample consisted of 25,004 children. In this sample, 3,965(15.9%) children had fever in the two weeks prior to the survey. The mean age of the children was 27 months (SD = 17.2 months).

### Bivariate findings

Table 1 shows the associations between fever and the hypothesized predictors. All of the predictors were significantly related to the occurrence of fever except 'having bed net", "whether children under 5 slept under bed net previous night" and "type of bed net that child slept under". A total of 414(9.2%) children had fever in the North Central, 1,145(20.2%) in the North East, while 269(8.8%) had fever in the South West. (p < 0.0001). Three thousand and fifty one (16.8%) had fever in the rural areas compared with 914(13.3%) in the urban areas. (p < 0.0001). Of the 3,110 respondents who had bed nets in their households, 506(16.3%) children had fever, while 2,604(83.7%) did not. (p = 0.082). Of the 3,697 children who slept under bed nets, previous night, 580(15.69%) had fever compared with 425(17.3%) who did not. (p = 0.085). Furthermore, of the 1380 children who slept under treated net, 222(16.1%) had fever compared with 283(16.4%) who did not. (p = 0.82).

**Table 1:** Occurrence of Fever by Selected Variables

	Fever Occurrence in Child				
	Yes	No	Total	Chi	P value
**Region**					
North Central	414(9.2)	4066(90.8)	4480(100.0)		
North east	1145(20.2)	4512(79.8)	5657(100.0)	519.3	<0.0001
North West	1017(15.12)	5707(84.9)	6724(100.0)		
South east	542(25.2)	1606(74.8)	2148(100.0)		
South south	578(19.7)	2358(80.3)	2936(100.0)		
South West	269(8.8)	2790(91.2)	3059(100.0)		
**Total**	**3965(15.9)**	**21039(84.1)**	**25004**(100.0)		
**Place of residence**					
Urban	914(13.3)	5960(86.7)	6874(100.0)		
Rural	3051(16.8)	15079(83.2)	18130(100.0)		<0.0001
**Total**	**3965(15.9)**	**21039(84.1)**	**25004(100.0)**		
**Possession of bed net in household**					
No	3404(16.1)	17689(83.9)	21093(100.0)		
Yes	506(16.3)	2604(83.7)	3110(100.0)	0.035	0.0.85
**Total**	**3910(16.2)**	**20293(83.8)**	**24203(100.0)**		
**Children <5 yrs slept under bed net last night**					
No	425(17.3)	2027(82.7)	2452(100.0)	2.92	0.085
Yes	580(15.69)	3117(84.3)	3697(100.0)		
**Total**	**1005(16.3)**	**5144(83.7)**	**6149(100.0)**		
**Type of bed net child slept under**					
Treated net	222(16.09)	1158(.9)	1380(100.0)		
Untreated net	283(16.4)	1443(83.6)	1726(100.0)	0.054	0.82
**Total**	**505(16.3)**	**2601(83.7)**	**3106(100.0)**		
**Sex of child**					
Male	206816.3	10588(83.7)	12656(100.0)	4.47	0.034
Female	189715.4)	10451(84.6)	12348(100.0)		
**Total**	**3965(15.9)**	**21039(84.1)**	**25004(100.0)**		
**Wealth quintile of household**					
Lowest (poorest)	1106(17.04)	5385(82.96)	6491(100.0)		
Second(poorer)	978(16.6)	4903(83.4)	5881(100.0)	30.78	<0.0001
Middle	770(15.8)	4105(84.2)	4875(100.0)		
Fourth(richer)	651(15.4)	3585(84.6)	4236(100.0)		
Highest(richest)	460(13.1)	3061(87.0)	3521(100.0)		
**Total**	**3965(15.9)**	**21039(84.1)**	**25004(100.0)**		
**Age (months)**					
<6	274(9.42)	2635(90.58)	2909(100.0)		
6-11	566(19.96)	2270(80.04)	2836(100.0)		
12-23	1027(21.03)	3857(78.97)	4884(100.0)		
24-35	800(17.77)	3703(82.23)	4503(100.0)	263.67	<0.0001
36-47	681(14.32)	4703(85.68)	4754(100.0)		
48-59	558(13.06)	3713(86.94)	4271(100.0		
**Total**	**3906(16.17)**	**20251(83.8)**	**24157(100.0)**		

Row percentages presented

A total of 2,068(16.3%) male children had fever compared to 1,897(15.4%) females. (p = 0.034). More children had fever in the rural areas compared with the urban areas (16.8% and 13.3% respectively) (p < 0.0001). Children aged 6-11 months and 12-23 months reported more fevers (19.9% and 21% respectively) than other children (p < 0.0001). In addition, fever prevalence decreased significantly as wealth quintile increased. A total of 1,106(17.04%) children from the poorest (lowest wealth quintile) household had fever, 978(16.6%) from poorer households (second wealth quintile) had fever, while 460(13.1%) from the richest households had fever (p < 0.0001).

### Multivariate predictors of fever

After controlling for all the predictors simultaneously in a multi-level model, fewer predictors remained significant (Table 2). Only age, sex of child and type of place of residence predicted the occurrence of fever. Wealth index and all bed net variables dropped out as predictors. Respondents in rural areas were more likely to have fever than those in urban areas (OR = 1.27, p < 0.0001, 95%CI: 1.16, 1.41). Children aged seven months and above were more likely to have fever than those less than six months. (p < 0.0001) The female children were less likely to have fever than their male counterparts (OR = 0.923, p = 0.022, 95% CI: 0.859, 0.988). However, the significant association of fever with wealth index in the bivariable analysis disappeared in the multi level logistic regression analysis. The children in the highest wealth quintile (richest) appeared to be less likely to have fever than the poorest (OR = 0.898, p = 0.75, 95% CI: 0.837, 1.14), but this association did not reach statistical significance. The estimated between region variance was 0.22(SE = 0.113) giving an ICC of 0.1.

**Table 2:** Multilevel Logistic Regression analysis for determinants of childhood fever

	*Odds Ratios*	*P value*	95% CI
**Type of Place of Residence**			
Urban			
Rural	1.27	<0.0001	1.16 - 1.41
			
**Child's age**			
<6 months			
6-11	2.45	<0.0001	2.13 - 2.91
12-23	2.64	<0.0001	2.28 - 3.04
24-35	2.14	<0.0001	1.84 - 2.47
36-47	1.65	<0.0001	1.42 - 1.92
48-59	1.49	<0.0001	1.28 - 1.74
			
**Wealth index of household**			
Poorest			
Poorer	1.03	0.57	0.93 - 1.13
Middle	1.01	0.91	0.90 - 1.12
Richer	1.01	0.91	0.89 - 1.14
Richest	0.98	0.75	0.84 - 1.14
			
**Literacy status of mother**			
Not literate			
Literate	0.93	0.38	0.800 - 1.089

## Discussion

The overall prevalence of fever found in this survey is quite high when compared with the prevalence of fever in other SSA countries. Studies have shown that the prevalence of fever is less than 4.2% in the developed countries [2]. However similar fever prevalence has been reported in studies in other parts of the countries [15]. The regional analyses revealed that fever is more prevalent in the north central, followed by north east. A study on fever prevalence in northern Nigeria documented a prevalence of 56.9% among under fives seen in a primary health care center [16]. This pattern is also obtainable at both village and household levels especially among the children under the age of 5.

The analyses also showed that fever is higher in rural than urban areas. This may be associated with the high level of poverty in the rural areas. Poor people live in dwellings prone to mosquito proliferation. It has been shown that the characteristics of wall construction are associated with malaria prevalence [2]. In another study on rural-urban differential in maternal responses to childhood fever [17]; urban mother's responses to fever seem to be better than that of rural mothers. This might explain or corroborate the high prevalence of fever in the rural areas reported in this analysis.

The results of this analysis suggest that the possession of bed nets is not associated with fever. In addition, no association exists between "whether child slept under bed net and type of bed net child slept under". However, households that had bed nets reported less fever than those who did not though the difference did not achieve significance. This result is in line with other findings in other parts of Africa on the use of insecticide treated bed nets, and their effectiveness when studied on a population basis. A study conducted by Lengeler and Snow [18] showed that childhood mortality due to fever reduced significantly in areas where high percentage of children slept under bed nets. In another study on bed net use and malaria, those who used bed nets had better knowledge of malaria than non-users; however, this usage did not influence malaria morbidity at the household level [19]. The low rate of the possession of bed nets (which might be as a result of inability to afford them) might have accounted for the high prevalence of fever especially in rural areas in particular, and in villages where poverty is more pronounced. Preventive measures such as insecticide-treated bed nets are unaffordable to the poor if they must pay for them.

In the bivariate analysis, fever was found to be highest among the poor, followed by middle group, followed by the wealthiest group. On the basis of country-level data it was estimated that 57.9% of all deaths from malaria in the world in 1990 occurred among the poorest 20% of the world's population [20], but within the poor countries, there is evidence that the costs of malaria fall more heavily on the very poor. In Malawi, for instance, households with very low income carried a disproportionate share of the economic burden of malaria, with total direct and indirect cost of malaria consuming 32% of annual household income, compared with 4.2% among households in the low-to-high income categories [21]. In Tanzania, mortality in children younger than 5 years after acute fever was 39% higher among the poorest than in the least poor. The poorest (20%) of people in selected developing countries were as much as 2.5 times less likely to receive basic public health services as the least-poor [22]. Also, in India, malaria was linked to poverty, with an increase in the trend of malaria in states with stagnant economy [23].

## Conclusions

While, overall bed net possession was low, less fever was reported in households that possessed bed nets. Malaria control strategies and interventions should be designed that will target the poor and make an impact on poverty. The results suggest that regional factors in Nigeria may be more influential in the risk of developing childhood fever than risks related to household wealth status. The mechanism through which wealth may affect malaria occurrence needs further investigation.

## Competing interests

The authors declare that they have no competing interests.

## Authors' contributions

All the authors conceived the study. OO was the principal investigator for Nigeria. DP and DB were collaborators from John Hopkins School of Public Health and contributed to the revising of the manuscript. OY conducted the analysis, BA and OO, contributed to data analysis and interpretation. OY and BA prepared the initial draft. All authors contributed to the draft. All authors read and approved the final manuscript.

## References

[B1] World Health Organization Severe and complicated malaria Trans R Soc Trop Med Hyg 2000 94 suppl 11103309

[B2] Teklehaimanot A Mejia P Malaria and Poverty New York Academy of Sciences 2008 10.1196/annals.1425.037 18579874

[B3] Alaba AO Fosu A, Mwabu G Malaria in children: economic burden and treatment strategies in Nigeria Malaria and Poverty in Africa 2007 University of Nairobi Press, Nairobi 73 104

[B4] World Health Organization World Malaria Report 2005 Geneva, World Health Organization

[B5] Alaba AO Alaba O Malaria in rural Nigeria: implications for the Millennium Development Goals 2009 Report, African Development Bank

[B6] Alaba AO Alaba O Malaria in children: implications for the productivity of female caregivers in Nigeria 2002 Annual conference of the Nigerian Economic Society

[B7] Anyanwu JC Demand for Health Care Institutions' Services: Evidence from Malaria Fever Treatment in Nigeria African Development Review 2007 19 2 304 34 10.1111/j.1467-8268.2007.00163.x

[B8] World Health Organization The African Malaria Report 2006 World Health Organization

[B9] National Population Commission (NPC) (Nigeria) and ICF Macro 2009 Nigerian Demographic and Health Survey (NDHS) 2008 Abuja, Nigeria: National Population Commission and ICF Macro

[B10] Fisher AA Way AA The Demographic and Health Surveys program: An overview International Family Planning Perspectives 1988 14 15 19 10.2307/2947652

[B11] Gwatkin DR Rutstein S Johnson K Pande RP Wagstaff A Socio-economic differences on health, nutrition, and population HNP/PovertyThematic Group 2000 Washington DC: World Bank

[B12] Stata 10.0 Stata Corp 2005 College Station, TX, USA

[B13] Byrk AS Raudenbush SW Hierarchical linear models: Applications and data analysis methods 1992 Newbury Park: SAGE Publications

[B14] McCullagh P Nelder JA Generalized Linear Models 1989 2 Chapman and Hall, London

[B15] Salako LA Brieger WR Afolabi BM Umeh RE Agomo PU Asa S Adeneye AK Nwankwo BO Akinlade CO Treatment of childhood fevers and other illnesses in three rural Nigerian communities J Trop Paed 2001 47 230 238 10.1093/tropej/47.4.230 11523765

[B16] Ikeh EI Teclaire NN Prevalence of malaria parasitaemia and associated factors in febrile under 5 children seen in PHC centers in Jos, North Central Nigeria Niger Postgrd Med J 2008 15 65 69 18575475

[B17] Uzochukwu BSC Onwujekwe EO Onoka CA Ughasoro MD Rural-urban differences in maternal responses to childhood fever in South East Nigeria PLOS ONE 2008 3 3 e1788 10.1371/journal.pone.0001788 18335058 PMC2258001

[B18] Lengeler C Snow RW From efficacy to effectiveness: insecticide-treated bednets in Africa Bull World Health Organ 1996 74 pages 8789931 PMC2486924

[B19] Nuwaha F Factors influencing the use of bednets in Mbarara municipality of Uganda Am J Trop Med Hyg 2001 65 877 882 11791991 10.4269/ajtmh.2001.65.877

[B20] Gwatkin D Guillot M The Burden of Disease among the global poor 2000 Washington DC, The World Bank 10.1016/S0140-6736(99)02108-X 10470717

[B21] Ettling M Mcfarland DA Schultz LJ Chitsulo L Economic impact of malaria in Malawian households Trop Med Parasitol 1994 45 74 79 8066390

[B22] Malaney P Spielman A Sachs J The malaria gap Am J Trop Med Hyg 2004 71 2 Suppl 141 146 15331830

[B23] Sharma VP Malaria and Poverty in India Current Science 2003 84 513 515

